# 32 × 32 Pixelated High-Power Flip-Chip Blue Micro-LED-on-HFET Arrays for Submarine Optical Communication

**DOI:** 10.3390/nano11113045

**Published:** 2021-11-12

**Authors:** Tae Kyoung Kim, Abu Bashar Mohammad Hamidul Islam, Yu-Jung Cha, Joon Seop Kwak

**Affiliations:** Department of Energy Technology, Korea Institute of Energy Technology, Ujeong-ro, 72, Naju-si 58330, Korea; tkkim@sunchon.ac.kr (T.K.K.); abmhis@kth.se (A.B.M.H.I.); yjrino@gmail.com (Y.-J.C.)

**Keywords:** micro light-emitting diodes (μ-LEDs), high electron mobility transistor (HEMT), flip-chip, solder bump bonding, μ-LED arrays

## Abstract

This work proposes the use of integrated high-power InGaN/GaN multiple-quantum-well flip-chip blue micro light-emitting diode (μ-LED) arrays on an AlGaN/GaN-based heterojunction field-effect transistor (HFET), also known as a high electron mobility transistor (HEMT), for various applications: underwater wireless optical communication (UWOC) and smart lighting. Therefore, we demonstrate high-power μ-LED-on-HEMT arrays that consist of 32 × 32 pixelated μ-LED arrays and 32 × 32 pixelated HEMT arrays and that are interconnected by a solder bump bonding technique. Each pixel of the μ-LED arrays emits light in the HEMT on-state. The threshold voltage, the off-state leakage current, and the drain current of the HEMT arrays are −4.6 V, <~1.1 × 10^−9^ A at gate-to-source voltage (*V_GS_*) = −10 V, and 21 mA at *V_GS_* = 4 V, respectively. At 12 mA, the forward voltage and the light output power (LOP) of μ-LED arrays are ~4.05 V and ~3.5 mW, respectively. The LOP of the integrated μ-LED-on-HEMT arrays increases from 0 to ~4 mW as the *V_GS_* increases from −6 to 4 V at *V_DD_* = 10 V. Each pixel of the integrated μ-LEDs exhibits a modulated high LOP at a peak wavelength of ~450 nm, showing their potential as candidates for use in UWOC.

## 1. Introduction

III-nitride compound semiconductors are very promising candidates for use in light-emitting diodes (LEDs) [1] and high electron mobility transistors (HEMTs) [2]. LEDs have been widely used in smart lighting applications such as solid-state lighting, micro displays, visible light communications, adaptive headlights for vehicles, and photo dynamic therapy [3,4,5,6]. Currently, underwater wireless optical communication (UWOC) is receiving significant attention with regard to the development of underwater navigation, sensor networks, and real-time underwater digital video streaming [7,8,9]. One of the most important parts of UWOC is the light source. The blue spectrum has a relatively small water attenuation [7,9]. High-power blue LEDs are a more suitable light source than lasers or laser diodes due to their low cost and their being less harmful to human eyes [7]. Therefore, high-power LEDs with a driving circuit are required for implementing these applications, where the driving circuit plays crucial role in improving the performance of an LED. On the other hand, traditional LED drivers suffer from a low efficiency and high power consumption due to the increase in parasitic elements caused by bonding wires [10]. The issues of parasitic elements can be circumvented by integrating LEDs with on-chip drivers [10,11,12]. AlGaN/GaN-based field-effect transistors (FETs) are GaN-based drivers that are very suitable for integrating GaN-based LEDs for high-power and high-frequency switching applications owing to their high breakdown voltage [13], wide range of operating temperatures [14], high operating frequency and speed [15], low specific on-resistance (*R_on_*) [4,10,15], and low power loss [16].

The on-chip interconnection between LED and HEMT can be formed using monolithic integration [2,3,4,5,10,12,13,17,18,19,20,21,22,23] and the flip-chip bonding technique [24,25]. Both technologies have their pros and cons. The on-chip monolithic integration of GaN-based LEDs with high-power electronic devices was demonstrated using the selective epi removal (SER) process [2,3,20,22], selective epitaxial growth (SEG) process [5,17,21,23], and a combination of the SER and SEG processes [12], where the LED-on-HEMT monolithic integration uses either with-wire [2,3,4,17,18,20] or without-wire [5,10,12,13,19,21] connections. The degradation of integration-induced device performances was reduced by the flip-chip bonding technique [12,24]. However, additional processes (substrate polishing and thinning) are required to complete the device fabrication, which results in an increase in the device fabrication complexity [25]. During SER monolithic integration, inductive-coupled-plasma (ICP) etching damages the HEMT underneath and the p-GaN surface, which seriously degrades the performances of LED-on-HEMT devices [4,12,17]. Somehow, the proposed SEG process minimizes the limitations of the SER process [4,17]; nevertheless, it suffers from the reliable metal interconnections between the SEG epitaxial layer and the SiO_2_ mask [26], as well as the epitaxial growth temperature between InGaN/GaN-based MQWs and AlGaN/GaN-based HEMT [17]. Therefore, the use of a combination of SER and SEG processes was demonstrated to be effective for removing the drawbacks of the SER and SEG processes, showing the excellent off-stage characteristics of HEMT and minimizing the number of parasitic components involved [12]. Unfortunately, fabricated LED-on-HEMT devices can only be implemented in low-power applications due to the low light output power (LOP) at a gate-to-source voltage (*V_GS_*) of 1 V [12]. To address these issues, a flip-chip solder bump bonding technique that is effective between LED and AlGaN/GaN-based (HEMT) will provide a better solution for the monolithic integration and improve the performances of LED-on-HEMT devices.

In this study, integrated μ-LED-on-HEMT arrays were created by interconnecting 32 × 32 pixelated flip-chip blue μ-LED arrays with 32 × 32 pixelated HEMT arrays; then, the performance of the μ-LED-on-HEMT device was investigated. The drain current, threshold voltage (*V_th_*), off-state leakage current, output characteristics, and transfer characteristics of HEMT arrays and the current-voltage (*I–V*) characteristics, LOP, and peak wavelength of LED arrays were systematically studied in order to understand the change in the performance of μ-LED-on-HEMT arrays. The μ-LED-on-HEMT arrays have a very low off-state leakage current <10^−9^ A at *V_GS_* = −10 V. The LOP is about 2 times higher than that of the monolithic integrated LED-on-HEMT devices, meaning that these will be suitable for various novel applications.

## 2. Device Structure and Measurement Setup

**Figure 1:**
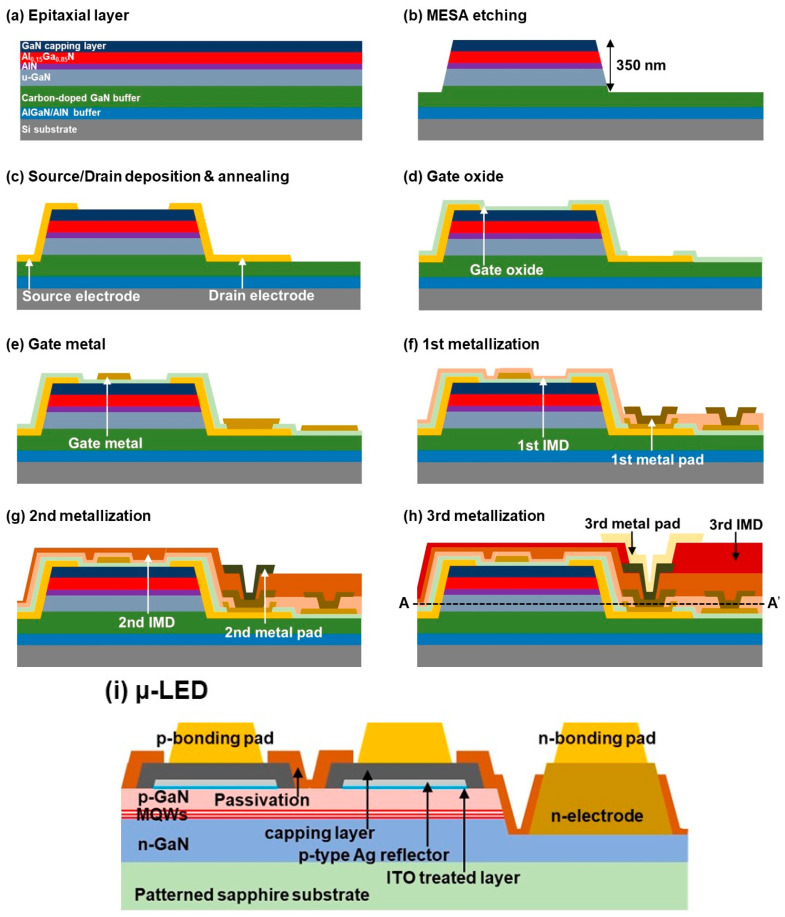
(**a**–**h**) Schematic fabrication steps of an HEMT device. (**i**) Basic epitaxial structure of a fabricated μ-LED.

AlGaN/GaN-based heterojunction field-effect transistors (HFETs), or HEMT devices, with a similar epitaxial structure were grown on Si(111) substrate using metal-organic chemical vapor deposition (MOCVD). The epitaxial structure consisted of a thin AlN seed layer, a 450 nm-thick AlGaN/GaN super-lattice buffer layer, a 4.5 µm-thick carbon-doped GaN buffer layer, a 250 nm-thick unintentionally doped GaN channel layer, a 1 nm-thick AlN barrier layer, a 21 nm-thick Al_0.15_Ga_0.85_N barrier layer, and a 2 nm-thick GaN capping layer, which were consecutively grown on the Si substrate as described in Figure 1a. After cleaning the wafer, photolithography was used for patterning the HEMT mesa structure, then it was selectively etched using ICP etching until a depth of 350 nm from the top was reached, as shown in Figure 1b. A Ti/Al/Ni/Au (20/80/50/50 nm) metal contact was deposited on the GaN/Al_0.15_Ga_0.85_N layer by electron-beam evaporation (EBE) to serve as source and drain electrodes, as described in Figure 1c. Generally, a thin GaN capping layer is used on the Al_0.15_Ga_0.85_N barrier in order to decrease the ohmic contact resistance. In order to make ohmic contact, the device was then annealed at 850 °C in ambient N_2_ for 1 min. A 100 nm-thick SiO_2_ gate oxide layer was deposited on the Al_0.15_Ga_0.85_N barrier by sputtering and then a lift-off process was carried out, as presented in Figure 1d. A Ni/Au (50/100 nm) metal gate was deposited on the SiO_2_/GaN channel and then the drain electrode layer was exposed by EBE, as described in Figure 1e.

**Figure 2:**
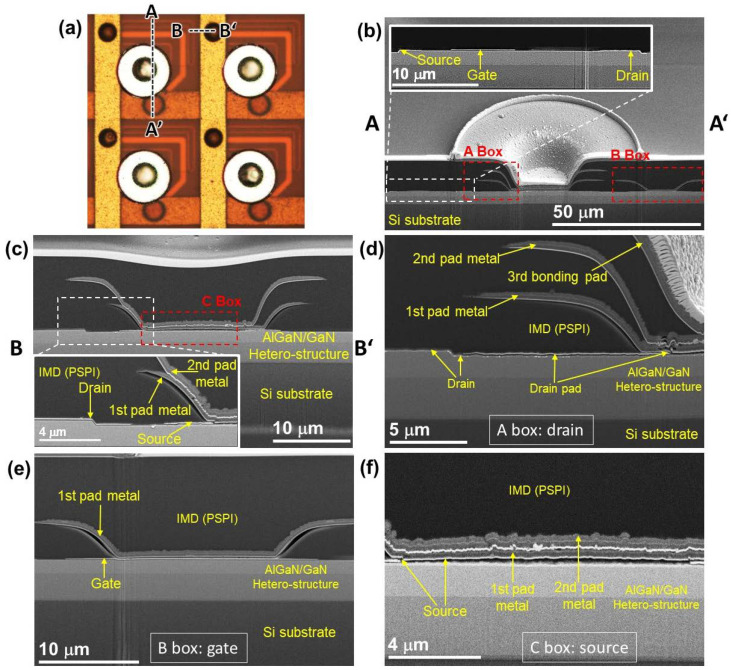
(**a**) Top-view optical microscope image of high-power HEMT arrays. High-resolution FE-SEM cross-sectional images of fabricated HEMT in the (**b**) AA’ and (**c**) BB’ directions according to the optical microscope image. Magnified FE-SEM cross-sectional images of the (**d**) drain, (**e**) gate, and (**f**) source metal pads, as marked by the boxes in (**b**,**c**), respectively.

A 1st 4 µm-thick organic photosensitive polyimide (PSPI) inter-metal dielectric (IMD) layer was formed and then patterned as via-holes on each source, drain, and gate electrode by photolithography. Using an oven furnace, the deposited 1st PSPI layer was cured for 60 min at 320 °C in ambient N_2_, followed by performing an O_2_ plasma treatment in order to improve the adhesion properties between PSPI and the deposited metal pad [27]. To form the 1st metal pad on each source and drain of the HEMTs, the via-holes of the 1st IMD layer were filled with a 1st metal stack of EBE-based Ni/Al (50/100 nm) followed by a sputtered Ti/Al/Ti/Al (30/500/30/500 nm) layer; then, a lift-off process was performed. The deposited 1st IMD and metal pad layers are presented in Figure 1f. A gate pad line on each HEMT device was also formed during this deposition. A 2nd IMD layer was then formed with a 4 µm-thick PSPI on the 1st metal stack. Via-holes were formed on the 2nd IMD layer in order to deposit a 2nd metal stack on the source and drain of each device, as shown in Figure 1g. The source pad line was then formed by filling the via-holes of the 2nd IMD layer. After that, a deposited 4 µm-thick PSPI 3rd IMD layer was patterned as via-holes on the 2nd metal stack of each drain. A drain metal pad was deposited by filling the via-holes with a 3rd metal stack, followed by a Ni/Au/Ni/Au (50/300/50/300 nm) drain bonding pad; then, a lift-off process was performed, as described in Figure 1h. The 2nd and 3rd metal stacks were similar to the 1st metal stack. The curing and plasma treatment conditions used for the 2nd and 3rd IMD layers were also similar to those used for the 1st IMD layer. The plasma treatment of PSPI is essential for preventing the metal from peeling off from the PSPI layer [27]. At an RF power = 30 W, PSPI plasma treatment was performed for 30 s in ambient O_2_. The PSPI layer was found to be more suitable compared to the SiO_2_/SiN_x_ layer due to its large dielectric constant, easy pattering by the standard photolithography process without any ICP etching, low cost made possible by reducing the number of complex fabrication processes necessary, and improved yield by minimizing the local stress [27,28]. The fabricated source, drain, and gate electrodes are also interactively described in Figure 2 using optical microscope and SEM images.

The blue LED wafers were grown on a c-plane patterned sapphire substrate (PSS) by MOCVD. The epitaxial structure consisted of a 30 nm-thick GaN buffer layer, a 4 μm-thick Si-doped n-GaN layer, a 100 nm-thick InGaN/GaN superlattice layer, a 41.5 nm-thick five pairs In_0.18_Ga_0.82_N/GaN multiple-quantum-well (MQW) layer, a 40 nm-thick Mg-doped p-Al_0.2_Ga_0.8_N electron-blocking layer, and finally a 200 nm-thick Mg-doped p-GaN layer, which were sequentially grown on the PSS. The μ-LED arrays with a pixel area of 115 × 115 μm^2^ were patterned using a photolithography process, then the mesa structure was etched by ICP in order to expose the n-GaN layer. The ITO treatment process was then performed on the p-GaN surface, which was described in our previous work [29]. A 250 nm-thick p-type Ag reflector was deposited on the ITO treated p-GaN surface. After that, a Ni/Al/Ni (100/500/100 nm) capping layer was deposited on the Ag/p-GaN surface; a Cr/Al/Ni (30/500/100 nm) n-type electrode was deposited on the exposed n-GaN surface; and finally a Ni/Au/Ni/Au (100/600/100/800 nm) capping layer was deposited on the n-electrode, which served as a bonding pad. The detailed structure and fabrication processes used for the 32 × 32 pixelated flip-chip μ-LED arrays are reported elsewhere [29,30]. A fabricated μ-LED with its epitaxial structure is shown in Figure 1i.

**Figure 3:**
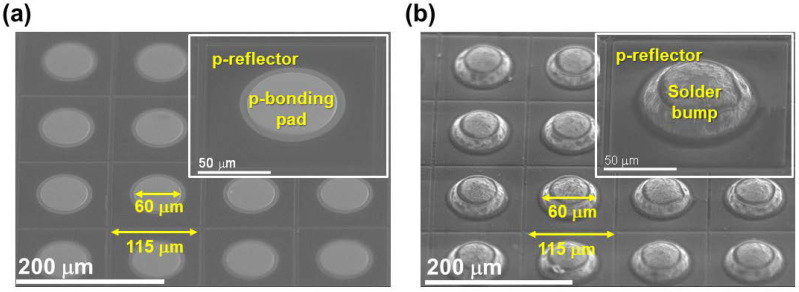
Top-view high-resolution FE-SEM cross-sectional images of fabricated µ-LED arrays (**a**) without and (**b**) with a 50 μm solder bump on the p-bonding pad. Inset shows without/with solder bump for one pixel.

In a fabricated μ-LED-on-HEMT device, each p-electrode bonding pad (see Figure 1i) of μ-LED arrays is connected with each drain electrode (see Figure 1h) of HEMT arrays using the flip-chip solder bump technique. First, a liquid soldering flux was deposited on each p-bonding pad of the μ-LEDs. A 25 μm-radius solder ball was formed on the top of liquid soldering flux. In order to form a solder bump, the μ-LED arrays were placed on the hotplate at 260 °C for 15 s. Next, the sample was added to ethyl alcohol in order to remove the flux solution from the p-bonding pad. Each solder bump/p-bonding pad of the μ-LED arrays was properly aligned with each drain bonding pad of the HEMT arrays; then, the μ-LED arrays were transferred onto the HEMT arrays. In order to form a proper electrical connection, the transferred device was placed onto a hotplate at 300 °C for 1 m and 30 s. Figure 3 describes the solder bump on each p-bonding pad of the μ-LED arrays. The common n-type bonding pad of the 32 × 32 pixeled μ-LED arrays was then connected with the ground (GND).

Keithley 2602B and 4145B (Cleveland, OH, USA) sourcemeters with a voltage sweep rate of 0.35 V s^−1^ were used for measuring the current-voltage (*I*–*V*) characteristics of the μ-LED and HEMT arrays, respectively. The electroluminescence (EL) intensity was measured by a Si p-i-n photodiode. The EL spectra and the peak emission wavelength of the μ-LEDs were recorded by a fiber-optic spectrometer (AvaSpec-2048, Avantes, Apeldoorn, The Netherlands). The absolute LOP vs. current (*L*–*I*) was measured by an integrating sphere system, which was connected to a spectrometer (CAS 140 CT, Instrument Systems, Munich, Germany). The light intensity distributions of the μ-LEDs were measured using a Metrolux beam monitor 8304. The cross-sectional and surface morphology images of the fabricated HEMT and μ-LED arrays were taken by an ultrahigh-resolution (HR) Schottky field-emission SEM (JEOL JSM-7610F Plus, Pleasanton, CA, USA).

## 3. Results and Discussion

**Figure 4:**
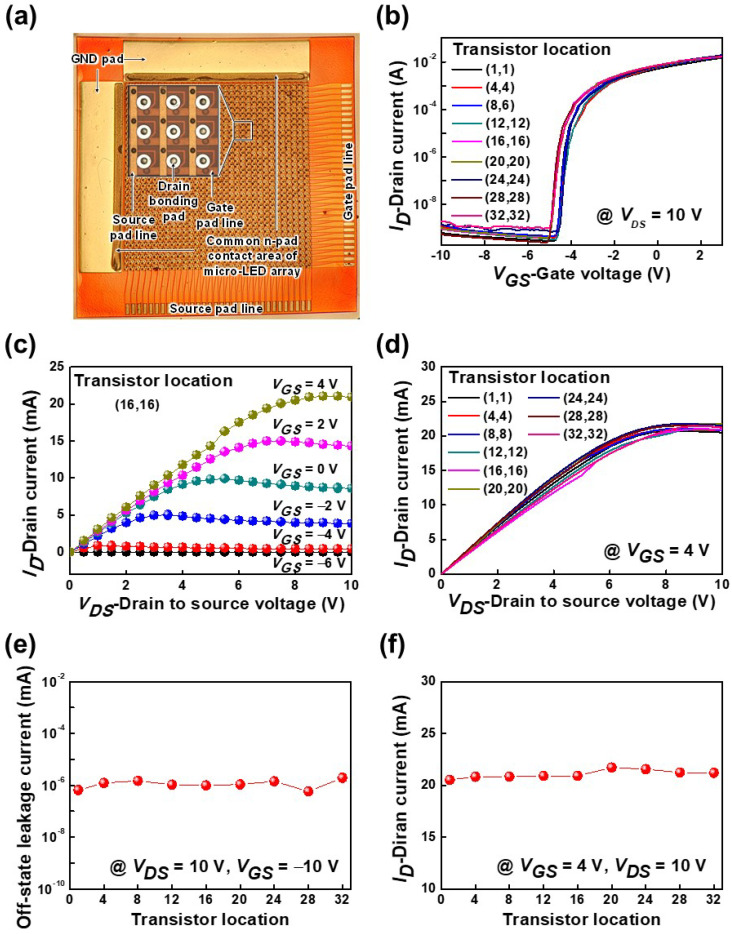
(**a**) Optical microscope image of 32 × 32 pixelated HEMT arrays. (**b**) Transfer characteristics of HEMT arrays for different pixel positions at *V_DS_* = 10 V. (**c**) Output characteristics for pixel location (16,16). (**d**) *I_D_* characteristics of HEMT arrays for different pixel locations at *V_GS_* = 4 V. (**e**) Off-state leakage current for different pixel locations at *V_GS_* = −10 V and *V_DS_* = 10 V. (**f**) Drain current for different pixel locations at *V_GS_* = 4 V and *V_DS_* = 10 V.

Figure 4a shows an optical microscope image of 32 × 32 pixelated HEMT arrays with drain and source bonding pads that are connected to the p-electrodes of μ-LED arrays and a supply voltage (*V_DD_*), respectively. The gate width, the source-to-gate, and the gate-to-drain distances of the fabricated HEMTs were 5, 5, and 12 μm, respectively. For a drain-to-source voltage (*V_DS_*) = 10 V, the transfer characteristics of the fabricated AlGaN/GaN-based HEMT arrays as a function of the gate-to-source voltage (*V_GS_*) are explained in Figure 4b. It was observed that the transfer characteristics of different pixels were fairly similar. The *V_th_* at which the drain current (*I_D_*) started to conduct was about −4.6 V for the fabricated HEMT arrays. Figure 4c shows the output characteristics as a function of the *V_DS_*. At *V_DS_* = 10 V (saturation region), the *I_D_* increased from 33.3 μA to 20.9 mA with the increase in *V_GS_* from −6 to 4 V. The *I_D_* characteristics were also similar for different pixel locations of HEMT arrays, as described in Figure 4d. Figure 4e reveals the measured off-state leakage current for different pixel locations, which was about 1.1 × 10^−9^ A at *V_GS_* = −10 V and *V_DS_* = 10 V. For different pixels, the measured *I_D_* was about 21 mA at *V_GS_* = 4 V and *V_DS_* = 10 V, as shown in Figure 4f. From the experimental results, we found that each pixel of the HEMT arrays had similar electrical characteristics.

**Figure 5:**
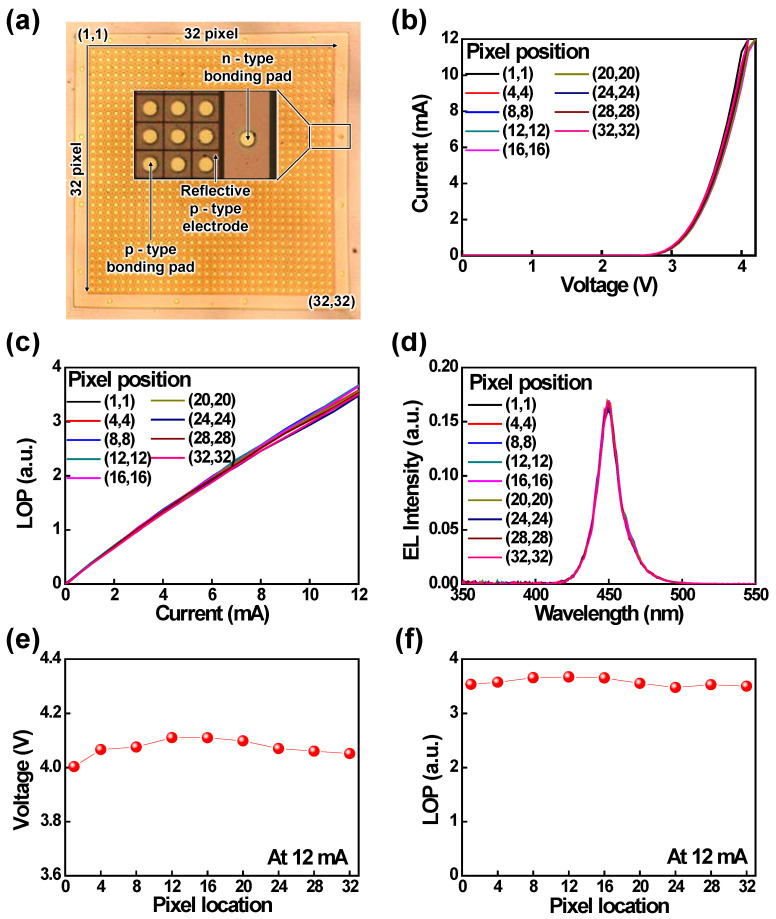
(**a**) Optical microscope image of 32 × 32 pixelated μ-LED arrays. (**b**) *I–V* characteristics, (**c**) LOP characteristics, and (**d**) EL spectra of the fabricated μ-LED arrays for various pixel positions. (**e**) Forward voltage and (**f**) LOP characteristics at 12 mA for various pixel positions of the fabricated μ-LED arrays.

Figure 5a shows the optical microscope image of fabricated 32 × 32 pixelated μ-LED arrays with p- and n-type bonding pads that are connected with drain and GND bonding pads, respectively, using flip-chip bonding technology. The *I*–*V* characteristics of different pixel positions are shown in Figure 5b; they are similar for each pixel of the μ-LED arrays. The forward voltage was ~2.9 V at 0.25 mA for each pixel of the μ-LEDs. The LOP and the EL spectra at 12 mA for various pixel positions of the μ-LED arrays are shown in Figure 5c,d, respectively; they were also similar for each pixel, similar to the *I*–*V* curve. At 12 mA, the forward voltages of different pixel locations are described in Figure 5e; they were about 4.05 V for the μ-LED arrays. Figure 5f shows the LOP characteristics for various pixel positions at 12 m; these positions were also similar (~3.5 mW) to the *I*–*V* and EL spectra curves. From the experimental results, it can clearly be observed that each pixel of the fabricated μ-LED arrays had a similar optoelectronic performance.

**Figure 6:**
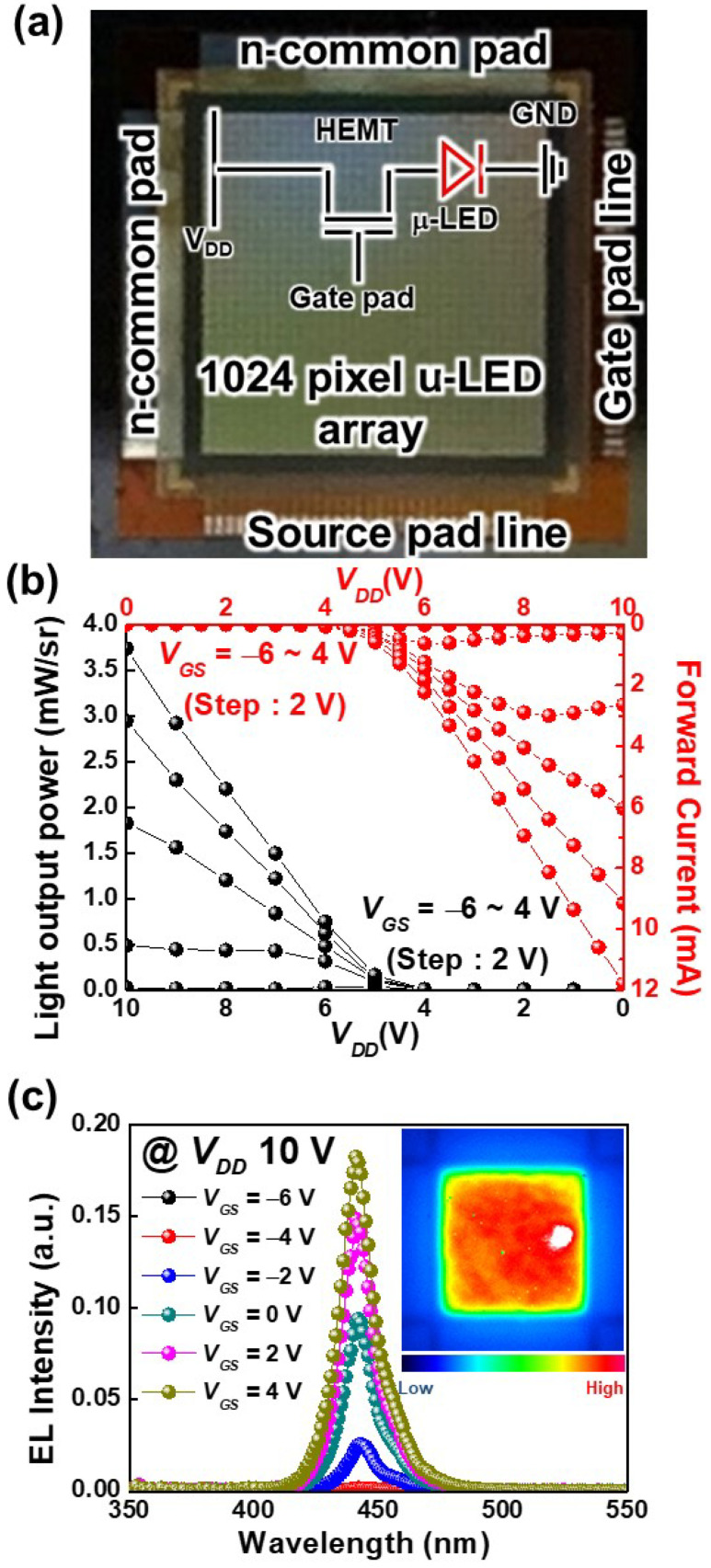
(**a**) Optical microscope image of the 32 × 32 pixelated μ-LED-on-HEMT arrays. (**b**) LOP and forward current characteristics of the μ-LED-on-HEMT arrays at different *V_GS_*. (**c**) EL spectra of fabricated μ-LED-on-HEMT arrays at different *V_GS_*. The inset shows the light intensity distribution at *V_GS_* = 4 V.

Figure 6 reveals the optoelectronic performances of the integrated 32 × 32 pixelated μ-LED-on-HEMT arrays. An optical microscope image of the integrated μ-LED-on-HEMT arrays with different electrodes is depicted in Figure 6a. The LOP and forward current of the μ-LED-on-HEMT device as a function of *V_DD_* is described in Figure 6b.

Both the LOP and forward voltage increased with an increase in *V_GS_*. At *V_DD_* = 10 V, the LOP and the forward voltage increased from 0 to 3.7 mW and 0 to 11.8 mA, respectively, as the *V_GS_* increased from −6 to 4 V. At *V_GS_* = −6 V, there was no LOP from the integrated μ-LED arrays with HEMT arrays. The *V_th_* of the HEMT arrays was about −4.6 V (see Figure 4); therefore, the integrated μ-LED devices could be operated at only ≥−4.5 V. In order to turn on the integrated μ-LEDs, the *V_DD_* should be greater than or equal to the turn-on voltage of the μ-LEDs (see Figure 5). It is worth noting that the LOP at *V_GS_* = 4 V increased eight times compared to the *V_GS_* = −2 V (0.5 mW), as the *V_DD_* was kept constant at 10 V. In addition, the on-state resistance decreased with an increase in *V_GS_* from −4 to 4 V due to the decrease in 2DEG resistance (this can be seen from the slope of the forward voltage characteristics) [24,31].

The EL spectra of the integrated μ-LEDs at different *V_G_s* are shown in Figure 6c. The increased *V_GS_* caused the forward current to increase, which resulted in an increase in the EL intensity. In addition, there was no shift in the peak emission wavelength with an increase in the *V_GS_*. The integrated μ-LEDs had peak emission wavelengths of 450 nm. The inset shows the uniform light intensity distribution over the chip size at a forward current = 11.8 mA, *V_GS_* = 4 V, and *V_DD_* = 10 V. The experimentally obtained optoelectronic performances of the integrated μ-LED-on-HEMT devices were comparable with the monolithically integrated LEDs with higher-power devices [3,12,17]. In addition, the LOP of the μ-LED-on-HEMT device was 3.5 mW at *V_GS_* = 4 V, which was two times higher than the reported LOP (1.5 mW) of the μ-LED source for UWOC [8]. This demonstrates that the integrated GaN-based μ-LED-on-HEMT device may be a promising candidate for UWOC and other lighting applications.

## 4. Conclusions

In summary, we successfully fabricated μ-LED-on-HEMT arrays and then investigated their optoelectronic properties. Firstly, we separately fabricated the Al_0.15_Ga_0.85_N/GaN-based 32 × 32 pixelated HEMT arrays on a Si wafer and the 32 × 32 pixelated flip-chip blue μ-LED arrays on PSS. Then, each p-type bonding pad of the μ-LED arrays was connected with each drain pad of the HEMT arrays by the flip-chip solder bump technique. The experimental results show that the electrical and optoelectronic performances of each pixel of the fabricated HEMT and μ-LED arrays were fairly similar. Thus, uniform optoelectronic performances were also obtained for each pixel of the integrated μ-LED-on-HEMT arrays. In order to turn on each pixel of the μ-LED-on-HEMT arrays, the *V_DD_* and the *V_GS_* voltages should be ≥3 and ≥−4.5 V, respectively. The *V_th_* of the fabricated HEMT arrays was about −4.6 V; at *V_GS_* = 4 V, the LOP increased eight times compared to the *V_GS_* = −4 V when the *V_DD_* was kept constant at 10 V. The optoelectronic performances of the μ-LED-on-HEMT device were comparable with those of other monolithically integrated LED-on-HEMT devices as well as the μ-LED light source of UWOC. Therefore, we anticipate that the on-chip interconnected μ-LED-on-HEMT arrays will be potential candidates for use in various novel applications.

## Data Availability

The data are available upon reasonable request from the corresponding author.
